# The first facile optical density-dependent approach for the analysis of doxorubicin, an oncogenic agent accompanied with the co-prescribed drug; paclitaxel

**DOI:** 10.1186/s13065-023-00976-5

**Published:** 2023-06-16

**Authors:** Ahmed Abdulhafez Hamad

**Affiliations:** Department of Pharmaceutical Analytical Chemistry, Faculty of Pharmacy, Al-Azhar University, Assiut Branch, Assiut, 71524 Egypt

**Keywords:** Optical density, Taxol, Doxorubicin, Paclitaxel, Degradation behavior, Eco-rating

## Abstract

Doxorubicin (DRB) is an anthracycline oncogenic drug extracted from cultures of Streptomyces *peucetius var. caesius*. It is frequently recommended as an anti-neoplastic agent for the treatment of diverse malignancies. It exerts its antineoplastic effect either via inhibiting the enzyme topoisomerase II and/or via intercalation to DNA or reactive oxygen species generation. In the present article, the direct, simple, one-pot, somewhat eco-safe, and non-extractive spectrophotometric system was executed to track doxorubicin, a chemotherapeutic remedy, in the presence of paclitaxel, a naturally occurring Taxan antineoplastic radical, through the greenness rated method. DRB’s optical density was studied in various mediums and solvents to develop the current approach. An acidic ethanolic solution was found to increase the optical density of the sample significantly. At 480 nm., the most remarkable optical density was obtained. Various experimental factors, including intrinsic media, solvent, pH, and stability time, were investigated and controlled. The current approach achieved linearity within the 0.6–40.0 µg mL-1 range, accompanied by a limit of both detection and quantification (LOD and LOQ) of 0.18 and 0.55 µg mL-1, correspondingly. The approach was validated under the ICH guidelines (Quality Guidelines). The system’s greenness and enhancement degree were estimated.

## Introduction

**Fig. 1:**
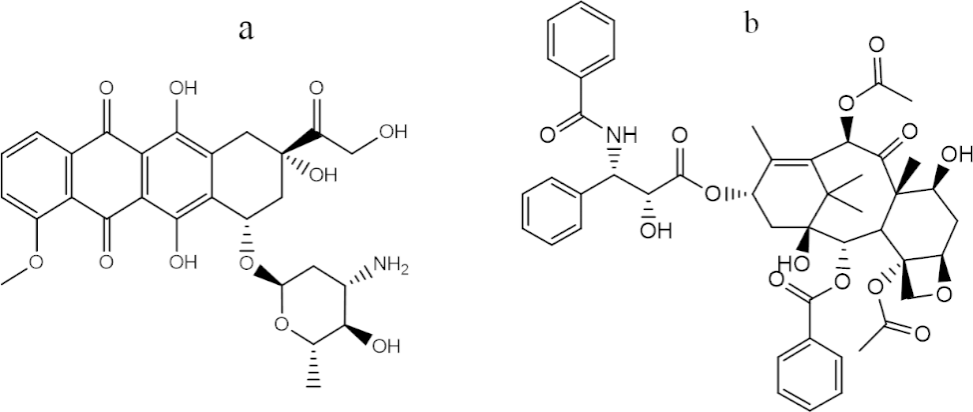
Structure of the studied coupled drugs; DRB (**a**) and PTX (**b**)

Doxorubicin (DRB, Fig. 1a) is an anticancer drug generated by the *Streptomyces peucetius var. caesius. Fungus*. It is frequently utilized in chemotherapy treatment for various cancers, including leukemia, Ewing’s sarcoma, Hodgkin’s disease, testicular, Kaposi’s sarcoma, several forms of carcinoma, breast, and tissue sarcomas [1]. The anthracycline moiety of DRB intercalates into the DNA double helix for impeding DNA attacking process by stopping the advancement of the topoisomerase II enzyme [2–4].

Another way in which doxorubicin is thought to affect cancer cells is by generating free radicals called Reactive Oxygen Species (ROS) and allowing them to damage cellular membranes, DNA, and proteins [5]. Lipid peroxidation and membrane injury, Mutagenesis, oxidative stress, and apoptotic pathways of cell death are all potential outcomes of the generated reactive oxygen species [6].

As a result of dose-dependent cardiotoxicity, myelosuppression, and resistance to drugs, DRB’s clinical use is limited in cancer treatment. Paclitaxel (PTX, Fig. 1b) is a diterpenoid isolated from *Taxus brevifolia* bark. [7]. It is a mitotic inhibitor used in cancer chemotherapy to cure various cancers, like ovarian, breast, lung, cervical, and pancreatic cancer. Taxanes work by reversibly binding to tubulin to anchor microtubule complexes and stimulate microtubule polymerization. One of the most effective drug combinations for treating solid tumors involves anthracyclines and taxanes. Paclitaxel and doxorubicin are the most concurrently administered among these. The two pharmacological action mechanisms produce synergy when the two classes are coupled. The paclitaxel/doxorubicin combination appears as a good and efficient strategy in the first-line management of many solid cancers. Additionally, for individuals with incurable malignancies, systemic combination chemotherapy is the final option [8]. Monitoring DRB levels is crucial due to the relative above [9, 10]. Since DRB cytotoxic antibiotics are required for both chemical and clinical testing, it is necessary to quantify their concentration. Various methods were reported for evaluating DRB in raw or medicinal formulations. For instance, one way that has been described is a chromatographic strategy either using the UV-VIS detector [11, 12] or the fluorescence detector [13, 14]. In addition, other techniques were reported such as chemiluminescence [15, 16], electroanalytical techniques [17, 18], electrophoretic methods [19, 20], immunoassay [21, 22], and nanoparticle design systems [23, 24]. Conversely, a few spectroscopic-based methods were discovered in the literature, including Raman scattering [25–27] and spectrophotometric methods [28–30].

Additionally, it is imperative to emphasize that HPLC systems require substantial amounts of high-purity organic solvents, multiple steps for preconditioning sample procedures, and costly apparatus and detectors. Furthermore, the decreased sensitivity of the previously documented spectrophotometric methodologies, as well as the tedious and complicated procedures that necessitated heating, extraction, and the use of more complex materials [31, 32], were obvious limitations. Therefore, it is crucial to create a spectrophotometric method that is green, direct, fast, simple, and sensitive for the analysis of the antineoplastic medication doxorubicin. The aforementioned objectives may be attained through the utilization of the spectrophotometric methodology, coupled with the inherent self-absorption properties of the investigated medication in an acidic ethanolic setting.

The suggested approach was thoroughly tested based on ICH criteria [33] (Quality Guidelines) and was successfully applied to promptly detect DRB in raw powder, medications, and human bodily samples. Only a few spectrophotometric [28–30] approaches for quantifying DRB in crude powder and marketable pharmaceutical preparations have been reported, and no colorimetric stability studies have been published.

Furthermore, there are no spectrophotometric methods for determining DRB, an anthracycline radical in the presence of a taxane, paclitaxel drug in bulk, vial, or other matrices. Therefore, it is essential to develop quick, sensitive, precise, and simple spectroscopic methodologies for determining DRB oncogenic agents. In conclusion, creating an accessible, trustworthy, fast, and highly delicate logical technique for estimating DRB was critical. The preceding target could potentially be achieved through the utilization of a spectrophotometric methodology, which boosts the intrinsic absorbing capacity of DRB by examining the impact of various diffusion media. The present study aimed to develop and authenticate an eco-friendly spectrophotometric method that is both selective and sensitive, while also being straightforward and expeditious, for the purpose of quantifying DRB. There is no longer a toxic organic solvent involved in the suggested analytical strategy.

Without impact from the sampling process or co-formulating ingredients, the current method was designed and validated in accordance with ICH requirements [34] to rapidly test DRB in unprocessed powder and prescription medications with exceptional accuracy and precision. The suggested approach was extended to further investigate DRB’s intrinsic stability under difficult environments, such as protogenic, oxidizing, and photolytic (UV, daytime, and direct sunlight).

## Experimental

### Apparatus

A Shimadzu (model UV-1601 PC) UV-visible spectrophotometer was used to conduct spectrophotometric tests (Tokyo, Japan). Quartz sample cells were used to measure the absorbance (1 cm for each). The control solutions were prepared using the Adwa AD11P pH meter. Thermostatic water-bath device (MLW type) made by Memmert GmbH in Schwabach, Germany, for heating. The mixer of superior quality is manufactured by industrial GEMMY, based in Taiwan; R.O.C. was applied for mixing purposes. Additionally, a SONICOR (model SC-101TH) was used. A sample centrifuge apparatus manufactured by Bremsen ECCO in Germany. The weighing process was conducted using a single-pan digital balance (Precisa XB 220 A, Switzerland). A water distiller for producing operational distilled water (TYUMEN-MIDI-A0-25 MO, Russia).

### Chemicals and materials

All solvents consumed during this investigation were of analytical purity. DRB (purity, 99.06%) and PTX (purity, 99.5%) were collected from Hikma Specialized Pharmaceutical Company (Industrial Badr City, New Cairo, Egypt). Adricin^®^ vial claimed to contain 50 mg of DRB (10 mg/5 mL), and Unitaxel^®^ injection containing 100 mg/16.67 mL were gifted from the previous company.

Tween-80, CMC Na (sodium carboxymethyl cellulose), and SDS (sodium dodecyl sulfate), all were dispersed as 1.0% v/v or w/v aqueous solutions and supplied by El-Nasr Chemical Co., (Cairo, Egypt). Other chemicals such as β-CD (β-cyclodextrin), CTAB (cetyl trimethyl ammonium bromide), and PVP (polyvinylpyrrolidone) were collected from Sigma-Aldrich (St. Louis, MO, USA) and also dispersed as 1.0% w/v aqueous solution.

Methyl alcohol, Ethyl alcohol, Acetonitrile, Acetone, DMF, Dioxane, HCl acid, NaOH, and hydrogen peroxide (30% v/v) were supplemented by El-Nasr Chemical Co. (Cairo, Egypt).

Britton-Robinson, the pH-adjusting solution [35–37], in the range of 2.0–12.0 pH was utilized.

### Stock solutions

Dissolving 10.0 mg of DRB in 100 mL of 100% ethanol in a calibrated volumetric flask labeled at 100.0 mL yielded a typical parent ethanolic solution. The stock standard solution was diluted with 100% ethanol to make a standard operating solution. Additionally, paclitaxel was made in the same manner. The solution containers were wrapped in aluminum foil and stored at a temperature of 4ºC to maintain the solution’s stability.

### General procedure steps

A series of volumetric adjusted flasks, each with a volume of 5.0 mL, was employed to hold samples of DRB operating solutions. Then 0.5 mL of 0.5 M HCl acid was added. Finally, the quantity was totalized up to the volume with absolute ethyl alcohol to achieve final DRB concentrations in the 0.6–40 µg ml^− 1^ range, and well mixed. After executing the identical steps lacking the drug to make a blank taster, the response was tracked at 480 nm. To develop a calibration graph, the final doses of DRB were plotted toward the comparable signal intensities.

### Investigation of the induced combination

The general analysis steps were applied for induced synthetic coupling to quantify the DRB at three concentrations (10, 15, 20, and 25 µg mL^− 1^) in the presence of PTX at a dose of 5 µg mL^− 1^. The ethanolic DRB and PTX solutions were transferred to the calibrated flasks in aliquots. Finally, HCl was moved to the solution, and the response was measured at 480 nm.

### Commercial product analysis

About 60.0 mL of absolute ethanol and a precise volume of the Santrone^®^ trade vial equivalent to 10.0 mg DRB were relocated into a 100.0-mL marked standardized flask. The flasks components were supplemented with absolute ethanol to attain a total volume of 100.0 mL for the DRB’s operating solution preparation. Various operating doses ranging from 10 to 25 µg mL^− 1^ were attained through additional solvent dispersion. The DRB samples underwent three rounds of analysis utilizing identical analytical steps.

### Stability examination

#### Stability examination in acidic and alkaline media

Using 0.1 M HCl or 0.1 M NaOH as a starting solution, 10.0 mg of DRB crude solution was diluted in 10 mL autoclavable fitted-capped tubes. Fluids were heated to 80 °C in a thermostatic water bath for different increasing periods. [28, 38, 39] Neutralization of each flask’s contents was performed using an opposite solution (0.1 M NaOH in case of acidic degradation and 0.1 N HCl in case of alkaline degradation), and the tubes were filled with absolute ethanol. Each alkaline and acidic medium’s triggered decomposition was built in complete darkness to eliminate any chance of light interfering with the degradation impact. As previously reported, the experiment was carried out with authentic fractions of 1 mL of the final solutions being deposited into 10-mL validated standardized jars.

#### Oxidative stressed degradation

Experimentation with heat deterioration by oxidation was performed using 5 mL of DRB standard solution (100.0 µg mL^− 1^), which was subsequently filled with 3.0% v/v H_2_O_2_. The sample was maintained in a hot bath at 80 °C. An appropriate volume of this solution containing 10 µg ml^− 1^ was carefully moved to a 10-mL standardized flask at 15-minute intervals for each measurement. The same analytical approach was used to test the remaining unharmed intact DRB.

#### Photodegradation

##### UV degradation

To test the effect of UV irradiation on the DRB parent solution (10 µg mL^− 1^), a 10 mL well-fitted stoppered pot was fixed in a covered steel box at 0.2 m and subjected for a day to 254 nm UV-producing lamp. A blank specimen was prepared parallelly and wrapped in aluminum foil to exclude the light effect.

##### Natural light degradations

The experiment was repeated as described in “UV degradation,“ and the samples were exposed to direct sunlight but with a one-hour gap to test the residual intact medication in the presence of fragmented molecules. Also, the test was replicated as stated in the “UV degradation” paragraph, except DRB samples were subjected to daylight (but not direct sunlight) for three successful days (to reach a total time of 24 h.).

## Results and discussion

**Fig. 2:**
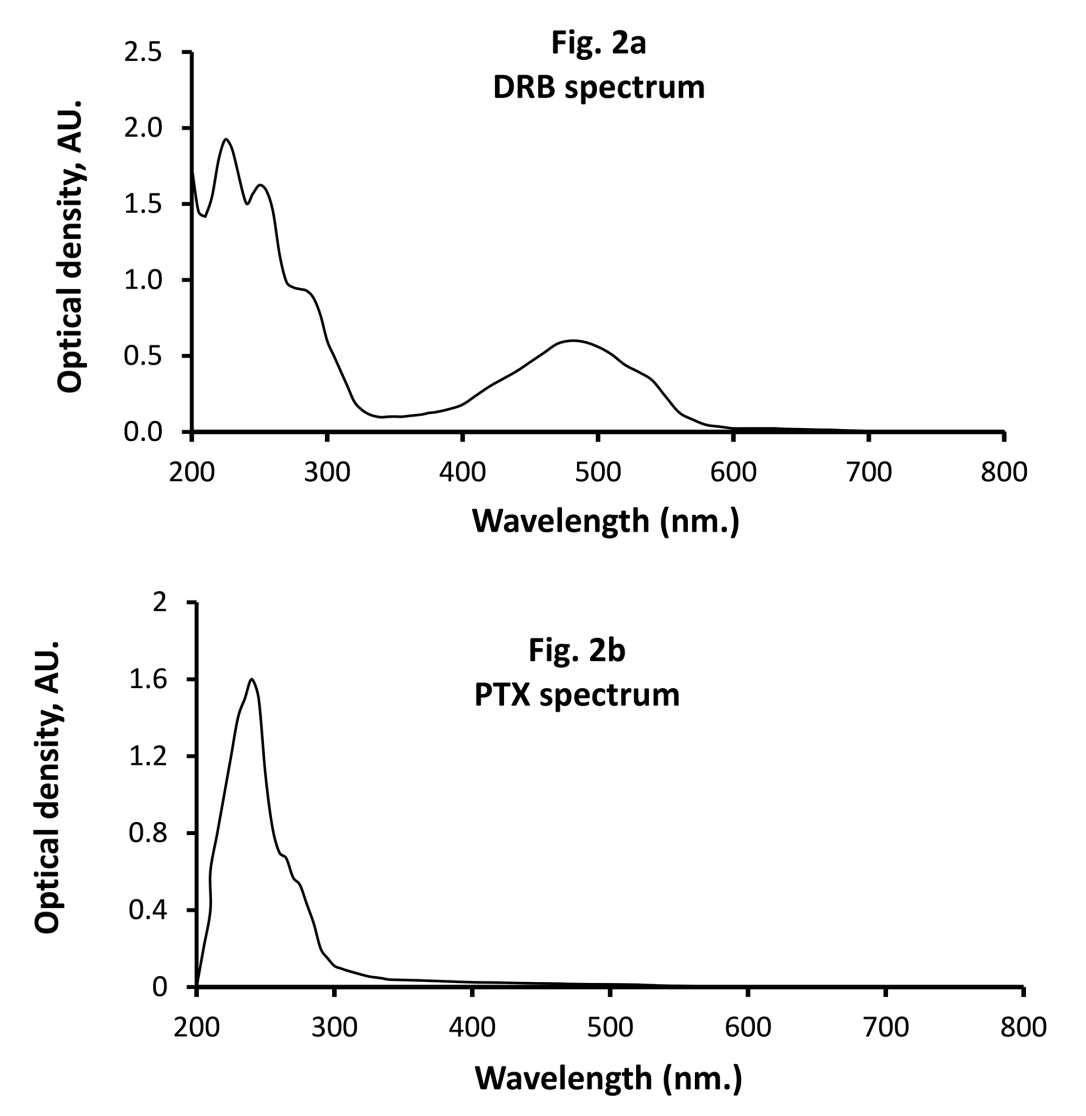
The UV-Vis spectra of DRB (**a**) and PTX (**b**)

The literature review indicates that several techniques and few spectrophotometric approaches have been documented for the determination of DRB in powdered form, branded prescribed drugs, and biofluids, with chromatographic methods being the most commonly applied. Nevertheless, these methodologies exhibit the drawback of necessitating costly solvents and apparatus. Consequently, the development of an environmentally friendly, cost-effective, sensitive, and facile procedure for detecting DRB in untreated powder and vials was considered necessary. The investigation employed spectrophotometry due to its advantageous features, including simplicity, selectivity, suitable sensitivity, and accessibility in most quality control laboratories. DRB was given a dark orange coloring because of its chemical structure, which includes the core of a conjugated anthracycline skeleton. Yet, in a solution containing water, its color is relatively weak. The utilization of different diluent and micellar media as drug matrices has emerged as a prominent approach to enhance the inherent absorbance and emission of several molecules. This technique has the potential to enhance sensitivity and consequently reduce the detection limit. The planned study utilized organic solvents that are low in hazard and safe for use, which have been observed to be associated with higher quantum yield [40]. The observed response augmentation could potentially be attributed to the stability or conservation of the stimulated singlet state [41]. Also, this increase in the micellar medium could be because unrestricted rotational motions are limited, which decreases the absorption or fluorescence [42]. Analogously, the aforementioned enhancement was observed in the absorption response relative to the enhancement observed while employing an organic liquid [39]. By employing this method, the spectrophotometric measurement of the suggested drug was greatly enhanced. Figure 2 illustrates the limited water absorption characteristics of DRB [43]. The spectroscopic properties of DRB were investigated using a number of organic solvents and surfactant-spiked media. The utilization of ethanol as an endogenous medium resulted in the most significant advancements in the fluorescence strength [39] and molecular absorptivity [43] of DRB in comparison to an aqueous solution. Consequently, ethanol was selected as an absorbance promoter to develop a novel spectrophotometric method for evaluating the referenced medication. The absorbance spectrum of DRB and PTX is depicted in Fig. 2a and b.

### Absorption Spectra of DRB

**Fig. 3:**
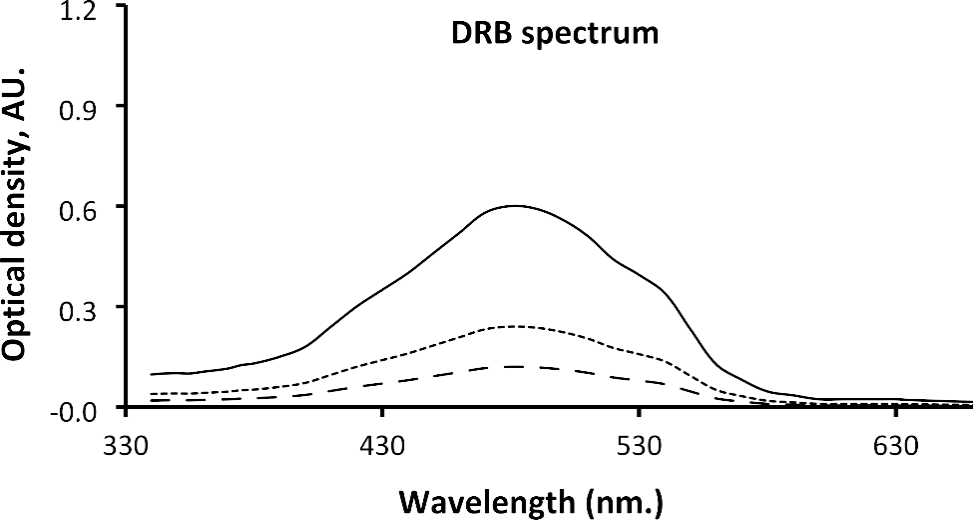
The UV-Vis spectra of the blank (− −), buffered DRB (20 µg mL^− 1^) in an aqueous system (….) and the ethanolic system (^_____^)

DRB absorbance spectra were monitored over the wavelength range from 200 to 800 nm. in aqueous, organized media systems and numerous organic solvents. As opposed to acidic aqueous systems, using acidic ethanol boosted DRB absorption by approximately 3.0 times. The spectra in Fig. 3 depict the absorption spectra of DRB in water and ethanol, both in the presence of 1.0 mL of 0.5 M HCl. The response strengthening appeared immediately following the finishing of the overall procedure and remained undetected for more than 2.0 h.

### Setting up experimental effectors

Many different experimental factors were tested and modified to see what effect they had on the DRB’s colorimetric features. There was an investigation on pH, buffer amount, surfactant type or organized matrix, dilution ratio of organic diluent, and time. To test the complete analytical method, only one variable was changed while the others remained the same.

#### The impact of operating medium

**Fig. 4:**
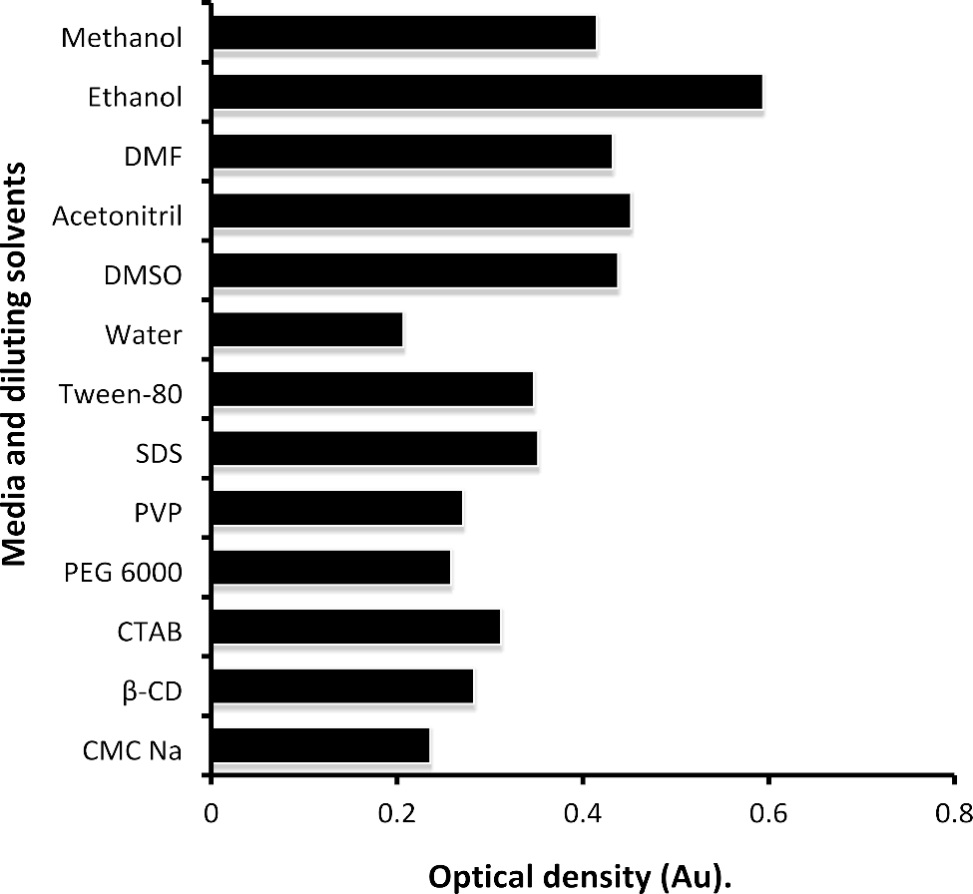
The impact of various media and solvents on the absorbance of DRB (20 µg mL^− 1^)

Different micelles were utilized to increase the absorbance of the DRB solution. Among these were the surfactants SDS (anionic), CTAB (cationic), PEG 6000 (non-ionic), Tween-80 (anionic), CMC^−^ Na^+^ (anionic polysaccharide), and other macromolecules like PVP and cyclodextrin. Using non-ionic surfactants, such as PVP and PEG 6000, does not influence the method’s absorption. On the other hand, CMC Na and CD; macromolecules have the same effect on the drug’s absorption. It turns out that the non-ionic surfactant, the anionic surfactant, and the cationic one all had a more considerable effect on the absorbance of the drug than water did (Fig. 4). The process of micellization has been observed to suffer from a delay in the presence of organic solvents. This phenomenon can be attributed to the complex relations between the hydrophobic interactions of long-chain hydrocarbon tails and the repulsive interactions of ionic head groups. Additionally, the influence of organic solvents on the aforementioned interactions further contributes to the observed delay [44].

The process of surfactant monomer aggregation occurs when the energy from the ionic head groups overcomes the attraction between their hydrocarbon part, thereby enabling the formation of a larger molecule. The static dielectric constant (ε) at 298.15 K for various substances such as dioxane, DMF, Ethylene Glycol, and DMSO, has been reported to be: 2.2, 38.3, 41.4, 46.7, and 80, respectively [45]. Given that the procedure involves adding more organic solvent, the repulsion between ionic groups is reduced, which lowers the response value measured in the mixed media. Because of this, micelle production in organic solvent/water; mixed settings may be slowed down. The transfer of hydrocarbon tails, methylene groups in the spacer, and methyl groups in the head part of surfactants to the micellar surface or interior becomes less thermodynamically favorable as the concentration of organic solvent increases in mixed organic solvent/water media. Rodríguez et al. reported that comparable outcomes were achieved through micellization of the 12-3-12 Gemini surfactant in diverse mixed solutions of water and organic solvents [46]. While surfactant CMC values increase in all solvent/water mixed media, they are significantly reduced when the percentage of organic solvent is increased to 10.0 [47].

#### Dispersing solvent impact

Methanol, acetonitrile, distilled water, ethanol, dimethyl sulfoxide, and dimethylformamide (DMF) were solvents utilized in the final dilution. A substantial increase in the molar absorption of DRB was observed when the organized medium and water were substituted with organic solvents. Ethanol was the most intense intrinsic absorbance, followed by methanol and DMF, with water having the lowest absorbance amplitude of all the studied solvents. It can be seen from the response data that the strength was inversely adequate to the polarity of the solvent utilized; and this is in agreement with the corresponding dielectric constants values of the investigated solvents, which were 46.7, 37.5, 36.7, 32.7, and 24.5 [48, 49]. The previous explanation matched with earlier published study of a similar design [39].

There are numerous ketonic and hydroxyl moieties in DRB. These groups tend to form hydrogen bonds with ethanol molecules, decreasing the non-radiative deactivation of an excited state by reducing the molecule’s intra-molecular dynamic, and increasing absorbance. In our study, we used ethanol as the sensitizing solvent because it enhanced absorbance more than any other solvent or medium (Fig. 4).

#### pH and volume impact

**Fig. 5:**
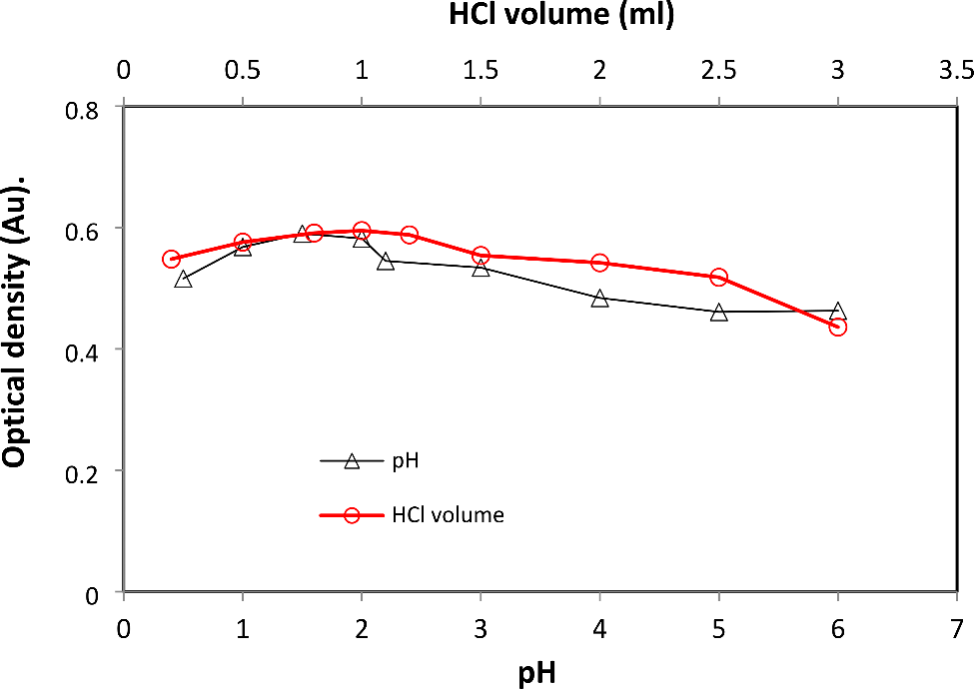
The impact of pH and HCl volume on the absorbance of DRB (20 µg mL^− 1^)

An investigation of DRB molar absorption in the employed medium was conducted. Experiments with hydrochloric acid (pH 1.0–2.0) and Britton-Robinson buffer solutions (pH 2.0–10.0) revealed that DRB’s intrinsic absorbance was pH-dependent. The original absorption of the drug was discovered to be pH sensitive. The pH 1.0–2.0 range showed the most remarkable improvement in the observed spectroscopic characteristics. According to Fig. 5, the spectroscopic property’s strength was reduced when the pH of the solution system increased or dropped. To get an ideal pH of 1.5, we used 1.0 mL of 0.5 M HCl solution. On the plus side, multiple volumes of 0.5 M HCl were tried to discover the optimal amount providing the highest value of measured characteristics in the presented procedures. 1.0 mL of HCl was employed to get the maximum molar absorption (Fig. 5).

#### Response stability and time

**Fig. 6:**
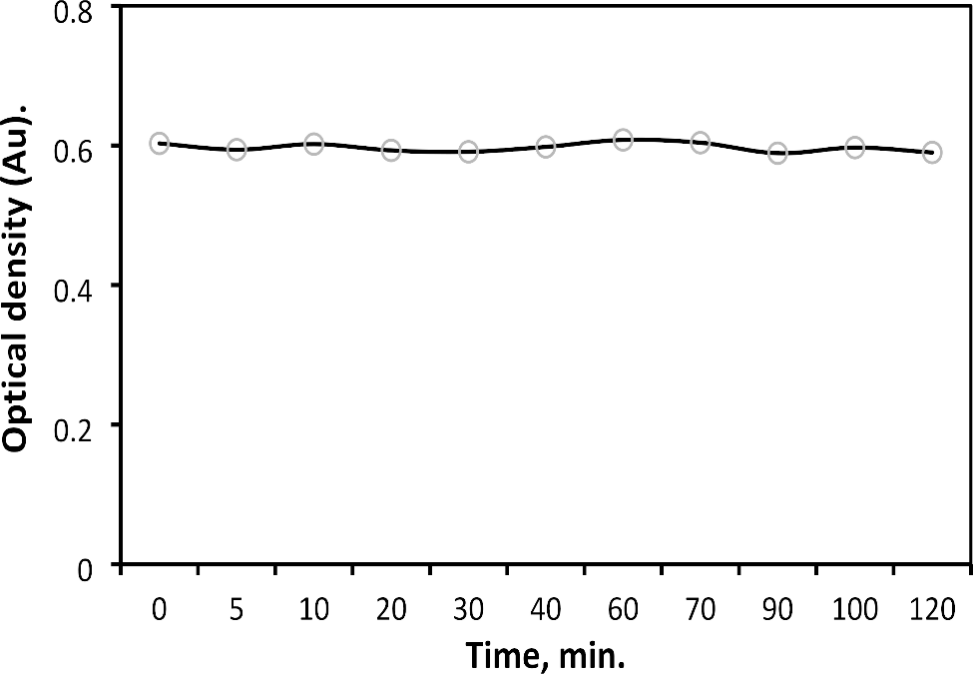
Effect of stability time on the absorbance of DRB (20 µg mL^− 1^) after procedure completion

Figure 6 shows that DRB’s molar absorptivity increased quickly after the procedure performing and remained steady for two hours.

### Validation of the method

The process of validation was conducted in obeying the guidelines established by the International Conference on Harmonisation (ICH) [33]. The study determined that the aspects of linearity, sensitivity, precision, accuracy, and resilience were found to be acceptable.

#### Calibration scale and method linearity

A number of standard DRB solution concentrations that encompass the linear range of DRB were used to demonstrate the linearity of the suggested technique. The study determined that the measured property of the method exhibited a linear correlation with the drug concentration when plotted over a concentration range of 0.6–40.0 µg ml-1. The strong correlation coefficients indicate the high degree of linearity of the method that has been established, as measured by the correlation coefficient (r). Table 1 presents crucial statistical factors for the procedure. The term LLOQ in analytical statistics means the minimum concentration of the calibration curve that can be accurately and precisely calculated. On the other hand, the greatest concentration on the calibration curve that could be identified with the necessary sensitivity was the ULOQ.

**Table 1:** Statistical parameter of the current approach

Parameter	Value
Linear range **(**µg mL^− 1^**)**	0.6–40.0
λ_max_	480 nm.
Slope	0.027
Intercept	0.038
Determination coefficient (r^2^)	0.998
Correlation coefficient (r)	0.999
Limit of quantitation **(**µg mL^− 1^**)**	0.55
Limit of detection **(**µg mL^− 1^**)**	0.18

#### Sensitivity limits

The calculation of LOD and LOQ was conducted in accordance with ICH recommendations, utilizing the formulas LOD = 3.3*S*_*a*_/*b* and LOQ = 10*S*_*a*_/*b*. The calibration curve slope is represented by the variable *b*, while the intercept standard deviation is denoted by *S*_*a*_. The proposed methodology exhibited a sensitivity of the examined drug with LOD and LOQ values of 0.18 and 0.55 µg mL − 1, respectively.

#### Accuracy

Five standard drug samples at different concentrations (5.0, 10.0, 15.0, 20.0, and 25.0 µg mL^− 1^) were estimated in triplets using the indicated approach. Table 2 presents the determined values for % recovery and standard deviation. The recovery percentages estimated exhibited a high degree of accuracy with a negligible percentage error (Er%). This indicates the accuracy of the built methodology.

**Table 2:** Accuracy assessment of the planned approach

Amount taken^c^	Amount found^c^	Rec*%	SD^*^	Er%
5.0	5.012	100.247	2.263	2.257
10.0	9.716	97.160	2.040	2.099
15.0	14.938	99.588	1.113	1.118
20.0	20.099	100.494	0.650	0.647
25.0	24.914	99.654	0.842	0.845

^c^ Concentration in (µg mL^− 1^)

^*^The value is the mean of three replicates measurements, SD; the standard deviation, and Er%; the relative error

#### Precision

The performance of the current approaches was determined through both intra-day and inter-day precision tests. For the given method, five different concentrations (5.0, 10.0, 15.0, 20.0, and 25.0 µg mL^− 1^) were determined for the present method evaluation at three distinct times on the same day.

The inter-day precision of the analytical process was evaluated by assessing the same three previous doses of medication over three successive days. Three separate samples of each dosage were tested for both precision levels. The data was validated through the utilization of percentage recovery and relative standard deviation. Table 3 displays low RSD% outcomes, which suggest the exceptional precision of the present methodology.

**Table 3:** Intra-day and inter-day precisions evaluation

Precision’s level	Conc. (µg mL^− 1^)	Rec%	Er%^b^	SD^b^
**Intra-day precision**	**5.0**	97.284	1.864	1.916
	**10.0**	99.383	2.410	2.425
	**15.0**	98.348	-1.165	0.284
	**20.0**	100.494	0.835	0.831
	**25.0**	98.012	-1.98	0.475
**Inter-day precision**	**5.0**	96.296	1.960	2.035
	**10.0**	100.494	1.864	1.855
	**15.0**	97.69	-2.305	0.79
	**20.0**	101.358	0.650	0.642
	**25.0**	97.95	-2.049	1.2290

^*^The value is the mean of three replicates measurements, SD; the standard deviation, and Er%; the relative error

#### Approach’s tolerance evaluation

The method’s capacity to adapt to intentional variations in the experimental setting was evaluated. Both the pH and volume of the solution were experimentally altered, while the other was kept constant. Table 4 indicates that there was no apparent modification in the recovery percentage or standard deviation. This unambiguously demonstrates the high level of reliability and resilience of the established protocol.

**Table 4:** Method’s tolerance evaluation

Parameter	Value	Rec%	SD
**pH of buffer**	+ 0.2	100.43	1.37
	ــ0.2	98.72	1.93
**HCl. Vol., mL**	+ 0.2	101.16	1.63
	ــ0.2	99.32	0.62

* Average value of three determinations, SD standard deviation, and Eris the relative error

#### Selectivity testing

A commercially available pharmaceutical formulation, adricin^®^ vial was employed to test the selectivity of the recommended technique. Due to the technique’s high percent recoveries, percent RSD, and percentage Er, classic DRB drug preparation ingredients did not influence this drug’s estimation.

Adding to its peculiarity, the synergistic interaction of DRB and the PTX medication was explored.

Due to the different absorbance regions on the UV-Vidivble wavelength scale (480 nm for DRB and 241 nm for PTX) of the two drugs (as previously shown in Fig. 2 (a&b), this approach yielded extremely acceptable findings when evaluating DRB in combinations without the impact of PTX.

At the bulk and dosage form levels, it’s crucial to keep in mind that the approach was devised. Percent recovery and RSD, which were found to be in the acceptable range at varied concentrations of DRB determination in sold form, imply that there was no evidence of interference (Table 5). As a result, the present methods can be utilized to identify DRB in the face of PTX.

**Table 5:** Tracking of DRB in the face of PTX, a concurrent drug in bulk and dosage form

matrix	^a^DRB+ PTX Taken	^a^DRB found	Rec%^b^	Er%^b^	SD^b^
**Bulk powder**	**10.0 + 5**	**9.74**	97.40	2.592	2.11
	**15.0 + 5**	**14.79**	98.60	-1.399	0.879
	**20.0 + 5**	**19.76**	98.82	1.172	1.065
	**25.0 + 5**	**24.58**	98.348	-1.165	0.284
**Dosage form**	**10.0 + 5**	**9.83**	98.39	1.604	0.301
	**15.0 + 5**	**15.09**	100.57	0.576	1.60
	**20.0 + 5**	**19.83**	99.19	-0.802	1.73
	**25.0 + 5**	**24.5**	98.00	1.99	2.128

^a^ Concentration (µg mL^− 1^)

^b^ Mean value of three determinations, Er is the relative error and SD standard deviation

### Application to pharmaceutical products

The advised approach was used to measure the drug dose in the achievable branded vial of DRB, such as the adricin^®^ vial [31]. The outcomes of the system were compared statistically through the student’s t- and F-tests (Table 6). According to the findings of both tests, the suggested and published methods’ precision and accuracy were inside the allowed values, which indicates that their results do not differ much. The recommended approach is more sensitive, easy, shorter required time, and uses no toxic or dying chemicals, making it superior to the previously published method. This study’s findings show that the developed method adequately measures the quoted medication with sufficient recoveries and no hindrance from the already present additives. As a result, the approach is a suitable alternative protocol for regular quality control and quality assurance testing of dosage formulations containing this drug.

**Table 6:** Analysis of vials using the proposed method and the published one ^[31]^

Parameter	Proposed methods	Reported method
**% Recovery** ^**a**^	98.21	99.58
**Standard deviation (SD)**	1.78	1.04
**Number of determinations**	5	5
**t- value** ^**b**^	1.34 (2.306)^b^	-
**F-value** ^**b**^	4.62 (6.338)^b^	-

^a^ The value is the average of five determinations for both the proposed methods and the reported one

^b^ Tabulated t and F values at 95% confidence limit

### Study of the drug stability

There were various destructive conditions in which DRB, the drug referenced in the article, was exposed. The current spectrophotometric approach determined the residual unaltered drug content following the degradation operation.

#### Degradation caused by alkali

**Fig. 7:**
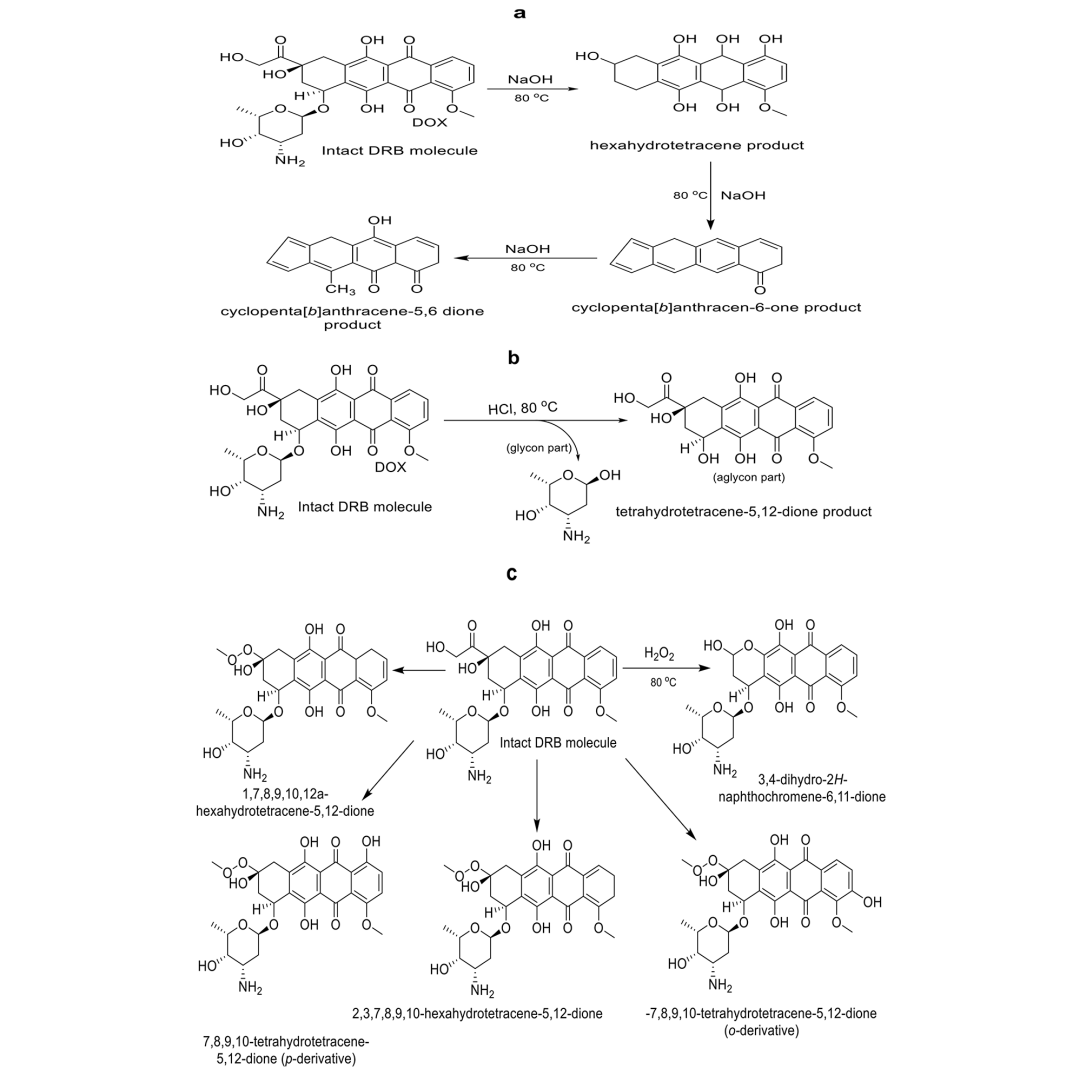
An alkaline (**a**), acidic (**b**), and oxidative (**c**) decomposition pathways for the DRB

Using a thermostatically controlled water bath, the studies found that the drug had significant potential for alkaline destruction, evidenced by a substantial drop in absorbance value after two hours of heating with a 0.1 M NaOH solution. After one hour, the medication had deteriorated, and the remaining intact drug percent was about 15% of its starting concentration under the circumstances mentioned above. Mass spectrometry detected four degradation products in a previous study [50]. However, the major and more stable one is illustrated in Fig. 7a, which depicts a potential DRB degradation pathway under various surrounding settings.

#### Degradation triggered by acid

It was found that DRB was rapidly susceptible to acidic solutions (Fig. 7b). When it was incubated at 80 °C [28, 38, 39] water bath to examine the effect of acidic conditions on its stability, about 76% of the starting dose remained unchanged after one hour of procedure application. In DRB, a glycosidic link connects the tetracyclic quinoid aglycone to the amino sugar. As with other glycosides, hydrolysis of doxorubicin produces the aglycone and amino sugar. [51].

#### Oxidative-triggered degradation

Adding 3% v/v H_2_O_2_ solution to the DRB solution for two hours at 80 °C reduced the absorption band slightly. After the first hour of exposure to the aforementioned oxidative environment, only 10.5% of destruction was seen in the DRB solution. An earlier work [38] found that oxidation might produce four breakdown products, as indicated in Fig. 7c. The benzene ring is hydroxylated at ortho- or para-position regarding the methoxy group, leading to peroxide production of this side chain and then eliminating the side chain [38].

#### Photolytic tolerance

The study results on the photodegradation of DRB have been highly varied. According to a few of these investigations, the medication is resistant to photolytic breakdown [38]. In contrast, some research has shown that the drug is light-sensitive [52–54]. This study’s findings demonstrate that DRB is extremely unstable when exposed to light. An aqueous solution of DRB deteriorated to 62.4% after exposure to indirect sunlight for ten hours. Similar solutions kept in the dark revealed a deterioration rate of 44.1% over the same period. Only 21.54% of the drug was eradicated in ethanolic solution during the same timescale.

Following these findings, one may deduce that daylight degradation of aqueous solutions can include both photo-degradation and hydrolytic effects.

A similar impact was seen when the drug solution was put in sunlight. It took two hours of exposure to sunlight for the drug to break down totally.

Within four hours of exposure to sunlight, around 73.6% of the drug had been destroyed in absolute ethanol. On the other hand, the DRB medication solution is resistant to UV radiation. It was found that a 37.7% deterioration rate was obtained after 10 h of UV irradiation.

In this investigation, DRB was shown to be relatively resistant to oxidizing agents and UV degradation. The hydrolysis process, particularly in an alkaline solution, and photolysis under direct sunlight were very prone to damage to the medication. As seen in Table 7, DRB degradation occurs under various conditions.

**Table 7:** The degradation study of the cited drug under different harsh conditions

Degradation type	Condition	Intact % found
**Acidic**	0.1 M HCl, 80 °C	76.5
**Alkaline**	0.1 M NaOH, 80 °C	14.8
**Oxidative**	3% (v/v) H_2_O_2_, 80 °C	89.5
**Photo-degradation**		
**In ethanolic medium**	UV radiation	92.3
	Direct sunlight	27.6
	Daylight	79.3
**In aqueous medium**	UV radiation	37.5
	Direct sunlight	26.5
	Daylight	38.5

### Ranking of the approach’s greenness

Biological and pharmaceutical analysts play a crucial role in protecting the environment and people from hazardous distribution media and organic residue [55, 56]. The field of green analytical chemistry is in a state of continuous development and necessitates regular consideration. The assessment of the environmental impact of analytical techniques has been carried out using contemporary measures such as the analytical eco scale score and the National Environmental Methods Index labeling [57]. These measures have been utilized to evaluate the degree of environmental friendliness of the analytical technique. An eco-scale was employed to assess the ecological impact of this approach. The evaluation outcomes of the eco-scale are determined by the deduction of penalty points or marks from a total of 100, which signifies an ideal green analysis [58]. The aforementioned markings indicate the hazards that were taken into consideration during the analytical approach. The higher the numerical value, the greater the worth of an analysis that exhibits a greater degree of environmental sustainability, as indicated by its “greenness” [59–61]. This is due to the fact that the developed method was experimented with ethanol as a solvent and required very little energy (less than 0.1 kWh) per sample and did not need extraction, heating, or other power-intensive operations. With a score of 90 on the eco-scale, the planned strategy was considered to be ecologically sound (Table 8).

**Table 8:** Method’s greenness evaluation using eco-scale

Item		Item PP score
Technique	Spectrophotometry	0
Reagent	HCl	1
Solvent	Ethanol	1
Temp.	Room temp	0
pH	Acidic	5
Energy	≤ 0.1 KWh/sample	0
Waste		3
Occupational hazards		0
Total penalty points (TPP)		10
Eco-scale total score: (100-PP)		= 90

Other green chemistry metrics such as the National Environmental Methods Index (NEMI), the Eco-Scale Assessment (ESA), the Green Analytical Procedure Index (GAPI), and the Analytical GREEnness measure (AGREE) are applied by analysts to evaluate the safety of analytical methods [62–64]. It has been shown that the proposed approach is safe for the environment and meets the (ESA score of 90). The AGREE rating is also quite high (0.86).

**Fig. 8:**
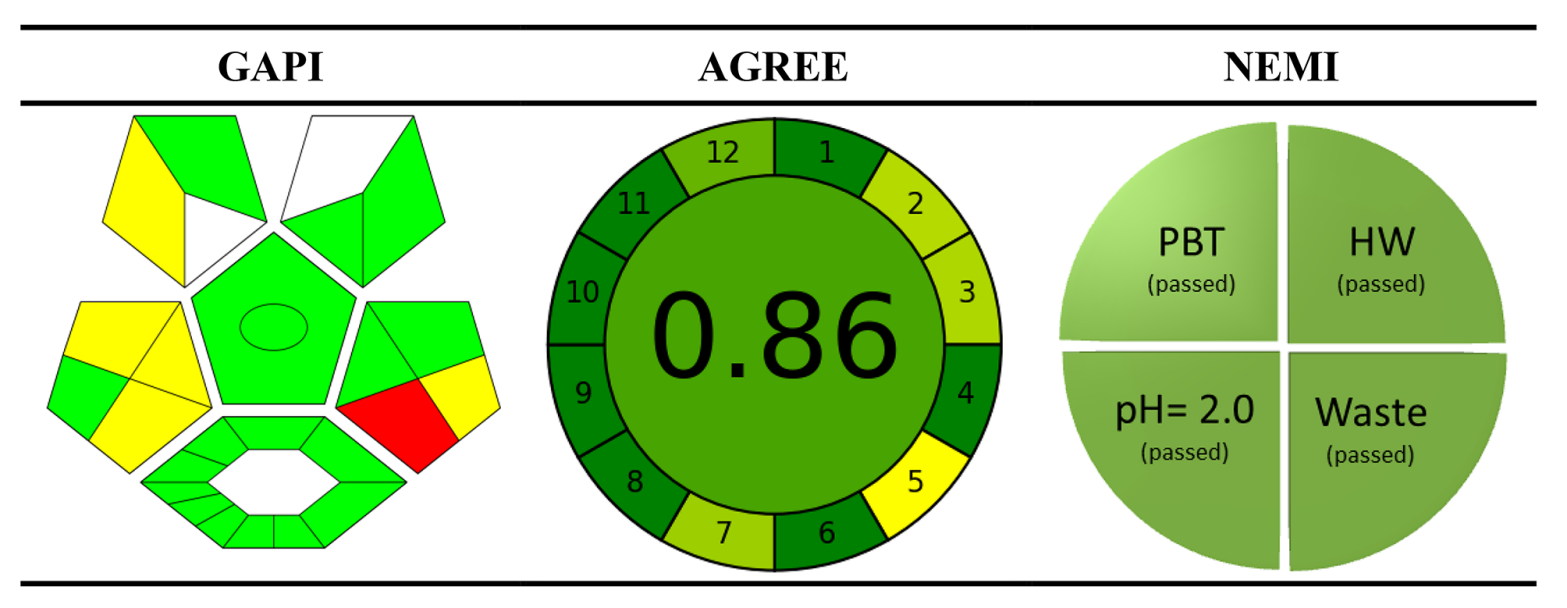
The greenness evaluation of the proposed method by applying different ecological metrics

The analytical method proposed in this study was evaluated using the aforementioned metrics (Fig. 8).

## Conclusion

The present methodology is characterized by its ease, speed, reproducibility, cost-effectiveness, and environmental friendliness, and does not necessitate complex processing steps, thermal treatment, or prolonged extraction procedures. In this approach, ethanol was employed as the system solvent due to its environmentally friendly properties and reduced potential for adverse effects. Furthermore, it should be noted that no solvents with harmful or carcinogenic properties are employed in this process. The method’s environmental sustainability is evidenced by its high eco-scale overall score of 90 out of 100. The proposed methodology exhibits advantages over numerous spectrophotometric techniques previously published for the assessment of the investigated medication. This is attributed to its notably enhanced sensitivity and reduced limit of quantification (LOQ), which enable the precise and accurate detection of the targeted drug in diverse matrices. The study demonstrated that the presence of PTX did not have an impact on Anthracycline (DRB) determination. Moreover, the spectrophotometric method is typically uncomplicated and cost-effective to employ. Furthermore, the proposed methodology may exhibit a degree of tolerance towards the coexistence of blends, typical additives, matrices, and alternative active constituents that could potentially be found in diverse forms of dosages.

## Data Availability

The datasets used and/or analyzed during the current study are available from the corresponding author on reasonable request.
